# Encorafenib, binimetinib, and cetuximab in *BRAF* V600E–mutated colorectal cancer: an early post-marketing phase vigilance study

**DOI:** 10.1007/s10147-022-02264-z

**Published:** 2022-11-10

**Authors:** Hidenori Sakata, Maki Murase, Takeshi Kato, Kensei Yamaguchi, Kenichi Sugihara, Shigenobu Suzuki, Takayuki Yoshino

**Affiliations:** 1 Department of Pharmacovigilance, Ono Pharmaceutical, Co., Ltd., 1-5, Dosho-Machi 2-Chome, Chuo-Ku, Osaka, 541-8526 Japan; 2 Department of Oncology Medical Affairs, Ono Pharmaceutical, Co., Ltd., 3-8-20, Marunouchi, Naka-Ku, Nagoya, Aichi, 460-0002 Japan; 3 Department of Surgery, National Hospital Organization Osaka National Hospital, 2-1-14, Hoenzaka, Chuo-Ku, Osaka, 540-0006 Japan; 4 Department of Gastroenterological Chemotherapy, Cancer Institute Hospital of the Japanese Foundation for Cancer Research, 3-8-31, Ariake, Koto-Ku, Tokyo, 135-8550 Japan; 5 Tokyo Medical and Dental University, 1-5-45, Yushima, Bunkyo-Ku, Tokyo, 113-8510 Japan; 6 Department of Ophthalmic Oncology, National Cancer Center Hospital, 5-1-1, Tsukiji, Chuo-Ku, Tokyo, 104-0045 Japan; 7 Department of Gastroenterology and Gastrointestinal Oncology, National Cancer Center Hospital East, 6-5-1, Kashiwanoha, Kashiwa, Chiba, 277-8577 Japan

**Keywords:** Colorectal cancer, Early post-marketing phase vigilance, Encorafenib, Binimetinib, Japanese patients

## Abstract

**Background:**

Triplet and doublet regimens of encorafenib plus cetuximab with and without binimetinib, respectively, were approved in Japan for unresectable, metastatic, *BRAF* V600E-mutated colorectal cancer (mCRC) that had progressed after 1–2 prior chemotherapies. This early post-marketing phase vigilance (EPPV) study collected adverse drug reactions (ADRs) from Japanese patients to ensure safety measures as appropriate.

**Methods:**

Patients with *BRAF* V600E mCRC who received the triplet or doublet regimens in Japan were selected for this study. ADRs were collected as spontaneous reports between November 27, 2020 and May 26, 2021. Serious ADRs were evaluated according to guidelines of the International Council for Harmonisation and the EudraVigilance list of Important Medical Event Terms.

**Results:**

An estimated 550 Japanese patients with mCRC received the triplet or doublet regimens during the 6-month EPPV period. Overall, 101 and 42 patients reported ADRs and serious ADRs, respectively. No ADRs leading to death were reported. The most frequently reported ADRs were nausea (17 patients), serous retinal detachment (16), decreased appetite (12), diarrhea (11), and vomiting (11). Among the important identified/potential risks that are defined in the risk management plans for encorafenib and binimetinib, eye disorder-related ADRs were observed in 32 patients, rhabdomyolysis-related ADRs in 12, hemorrhage-related ADRs in 7, and hepatic dysfunction-related ADRs in 7. Of 22 patients with serious eye disorders, 20 recovered or were recovering during the EPPV period.

**Conclusion:**

The safety profile in this EPPV study was similar to that from the phase III BEACON CRC study and no new safety concerns were identified.

**Supplementary Information:**

The online version contains supplementary material available at 10.1007/s10147-022-02264-z.

## Introduction

Tumors with the activating mutation V600E in *BRAF* account for 5–10% of colorectal cancers and are associated with a poor prognosis and a low response to epidermal growth factor receptor (EGFR; abbreviations are listed in Online Resource 1) inhibitors [1–4]. An open-label, international, phase III study (BEACON CRC) for patients with metastatic colorectal cancer with the *BRAF* V600E mutation that were previously treated with 1–2 prior chemotherapies demonstrated that both a triplet regimen of a BRAF inhibitor (encorafenib), a mitogen-activated protein kinase (MEK) inhibitor (binimetinib), and an EGFR inhibitor (cetuximab), and a doublet regimen of encorafenib and cetuximab significantly improved overall survival and the objective response rate with a tolerable safety profile compared with the investigators’ choice of either cetuximab and irinotecan or folinic acid, fluorouracil, irinotecan, and cetuximab [5]. The doublet regimen has been approved for unresectable advanced or recurrent colorectal cancer with the *BRAF* V600E mutation in the United States, the European Union, Japan, and Korea, while the triplet regimen has been approved for the same indication only in Japan. Combination therapy using encorafenib and binimetinib has also demonstrated favorable efficacy in patients with advanced melanoma with a mutation in *BRAF* V600 [6, 7], leading to its approval worldwide. While a manageable safety profile and a low discontinuation rate have been shown using encorafenib and binimetinib for advanced *BRAF*-mutated melanoma [8, 9], safety information for the triplet and doublet regimens of encorafenib plus cetuximab with and without binimetinib for *BRAF* V600E-mutated colorectal cancer has been limited in Japanese patients because only 3 and 6 patients were enrolled in Japan for evaluation of the triplet and doublet regimens, respectively, in the BEACON CRC study.

We conducted an early post-marketing phase vigilance (EPPV) study to collect reports of adverse drug reactions (ADRs) during the first 6 months after the approval of the triplet and doublet regimens for *BRAF* V600E-mutated colorectal cancer. The EPPV is a post-marketing pharmacovigilance surveillance study that was enacted by the Ministry of Health, Labour and Welfare of Japan to promote the appropriate use of newly approved drugs and regimens and to promptly accumulate their ADRs, especially those defined in the risk management plan (RMP). EPPV studies generally require no pre-registration but collect ADRs as spontaneous reports from healthcare professionals (HCPs) or patients. Here, we report the first real-world safety information on the use of encorafenib and binimetinib in patients with *BRAF* V600E-mutated colorectal cancer that was collected during the EPPV study period.

## Patients and methods

### Patients

Target patients in this EPPV were those with unresectable, advanced or recurrent, *BRAF* V600E-mutated colorectal cancer who received the triplet or doublet regimens in Japan. Because no pre-registration was required, the total number of the target patients receiving the triplet or doublet regimens during this surveillance period was estimated using a commercially available clinical database (Medical Data Vision Co., Ltd., Tokyo, Japan). In brief, the proportion of patients with malignant melanoma who received encorafenib in the database to those registered in a post-marketing all-case surveillance study was calculated. The number of patients with advanced or recurrent colorectal cancer who received encorafenib in this EPPV study was estimated using that proportion.

### ADRs

ADRs that were reported between November 27, 2020 and May 26, 2021 were collected as spontaneous reports from HCPs or patients and coded to MedDRA, version 24.0. ADRs were defined as adverse events if their causal relationships with encorafenib or binimetinib could not be ruled out. ADRs of particular interest were those listed in the RMPs for encorafenib and binimetinib (Online resource 2). Each ADR was graded as a serious ADR (SADR) or a non-serious ADR (non-SADR) according to the safety guidelines of the International Council for Harmonisation of Technical Requirements for Pharmaceuticals for Human Use. In brief, SADRs were ADRs that led to death, threatened life, led to persistent or significant disability or incapacity, required hospitalization or prolongation of existing hospitalization, caused a congenital anomaly or defect, and were considered medically important. Some non-SADRs were re-classified into SADRs, but not vice versa, according to the important medical event (IME) list that was developed by the EudraVigilance Expert Working Group of the European Medicines Agency to instantly identify an SADR. The number of SADRs and non-SADRs were counted individually, and therefore, an individual single patient could possibly be included in both categories of an ADR.

### Ethics

This EPPV was conducted in accordance with Good Vigilance Practice of the Ministry of Health, Labour, and Welfare of Japan, for which obtaining approval from the ethics committee of each institution and informed consent from each patient was not mandatory.

## Results

The number of patients with advanced or recurrent unresectable *BRAF* V600E-mutated colorectal cancer who received the triplet or doublet regimens during the 6-month surveillance period was estimated to be approximately 550. Overall, 101 patients had ADRs. Male and female patients with ADRs accounted for 43.6 and 55.4%, respectively; the sex of 1.0% of the patients was unknown. The ages were < 65 years in 54.5%, 65–74 years in 33.7%, ≥ 75 years in 10.9%, and unknown in 1.0% of the patients.

**Fig. 1:**
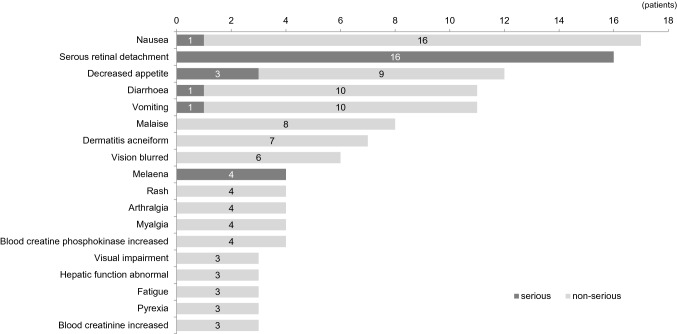
Adverse drug reactions observed in ≥ 3 patients. Regardless if their severity was initially reported as non-serious by health care professionals or patients, all cases of serous retinal detachment and melena were automatically graded as serious ADRs according to the important medical event list of the European Medicines Agency

Of 101 patients with ADRs, 42 patients had SADRs and no patients died due to ADRs (Fig. 1). The most common ADRs included gastrointestinal disorders, eye disorders, dermatological disorders, and pain. ADRs that were defined as or related to important identified risks and important potential risks in the RMPs were eye disorders in 32 patients (22 SADRs), Palmar-plantar erythrodysesthesia syndrome in 2 (0 SADRs), cardiac dysfunction in one (1 SADR), rhabdomyolysis in 12 (2 SADRs), hepatic dysfunction in 7 (1 SADR), hemorrhage in 7 (6 SADRs), interstitial lung disease (ILD) in one (1 SADR), renal impairment in 5 (2 SADRs), and venous thromboembolism in one (1 SADR) (Table 1).

**Table 1:** Adverse drug reactions listed in important risks of encorafenib and binimetinib

	Any	Serious
**Eye disorders-related ADRs**	**32**	**22**
Serous retinal detachment	16	16
Vision blurred	6	0
Visual impairment	3	0
Macular detachment	2	2
Angle-closure glaucoma	1	1
Retinal oedema	1	1
Subretinal fluid	1	1
Uveitis	1	1
Color blindness acquired	1	0
Dry eye	1	0
Visual acuity reduced	1	0
Vitreous floaters	1	0
Xanthopsia	1	0
**Palmar-plantar erythrodysesthesia syndrome-related ADRs**	**2**	**0**
Palmar-plantar erythrodysesthesia syndrome	2	0
**Cardiac dysfunction-related ADRs**	**1**	**1**
Cardiac failure	1	1
**Rhabdomyolysis-related ADRs**	**12**	**2**
Blood creatine phosphokinase increased	4	0
Myalgia	4	0
Blood creatinine increased	3	0
Renal failure	1	1
Renal impairment	1	1
**Hepatic dysfunction-related ADRs**	**7**	**1**
Hepatic function abnormal	3	0
Drug-induced liver injury	1	1
Alanine aminotransferase increased	1	0
Ammonia increased	1	0
Aspartate aminotransferase increased	1	0
Liver disorder	1	0
**Hemorrhage-related ADRs**	**7**	**6**
Melena	4	4
Gastrointestinal hemorrhage	1	1
Ulcer hemorrhage	1	1
Epistaxis	1	0
**Interstitial lung disease-related ADRs**	**1**	**1**
Interstitial lung disease	1	1
**Renal impairment-related ADRs**	**5**	**2**
Blood creatinine increased	3	0
Renal failure	1	1
Renal impairment	1	1
**Venous thromboembolism-related ADRs**	**1**	**1**
Deep vein thrombosis	1	1
Pulmonary embolism	1	1

Risk categories specified in the risk management plan are shown in bold, and adverse drug reactions in each category are shown in regular text

Eye disorders were the most common ADR, and serious eye disorders were observed in 22 patients. The median time to the first onset was 2 days (range, 1–97 days); 16 patients had eye disorders within one week, 12 within 2 days, 5 after one week, and the onset timing was unknown in 1 patient. It should be mentioned that these serious eye disorders were graded as SADRs according to the IME list but only 1 eye disorder (serous retinal detachment) was graded as a SADR in the spontaneous report, while the others were reported as non-SADRs. Of the 22 patients with serious eye disorders, 20 recovered or were recovering from the SADRs (Table 2). Serious eye disorders in 2 patients were not recovered at the date of data cutoff; however, no serious outcomes were recorded afterward. The most common eye disorder was serous retinal detachment in 16 patients. All patients with serous retinal detachment recovered or were recovering, of whom 6 continued with or without a dose decrease, while 10 patients interrupted or discontinued the treatment. The other patient with serous retinal detachment recovered after the date of data cutoff.

**Table 2:** Serious eye disorders in 22 patients

Adverse drug reaction	Outcome	*N*	Action taken	*n*
Serous retinal detachment	Recovered/recovering	16	Interrupted/discontinued	10
			Dose decreased	1
			Continuing (no dose change)	5
Subretinal fluid	Recovered	1	Interrupted/discontinued	1
Macular detachment	Recovered	2	Interrupted/discontinued	2
Angle-closure glaucoma	Not recovered	1	Interrupted/discontinued	1
Uveitis	Not recovered	1	Interrupted/discontinued^a^	1
Retinal oedema	Recovered	1	Interrupted/discontinued	1

When a patient experienced multiple onsets of an adverse drug reaction, only the action and outcome of the first onset of each patient were counted

^a^The doublet regimen, i.e., no binimetinib, had been administered in this patient

One SADR of ILD was observed in a male patient in his 70s. After a 2-week administration of the triplet regimen (encorafenib 300 mg once daily, binimetinib 45 mg twice per day, and cetuximab with an unknown dose), the patient reported difficulty breathing. Because a high serum KL-6 level of 545 U/mL, ground-glass opacity, and a negative β-D-glucan test were determined, the patient was diagnosed with drug-related ILD. The triplet regimen was interrupted, and an inpatient daily administration of prednisolone 50 mg was initiated. Intravenous methylprednisolone pulse therapy was also provided during the patient’s hospitalization. The patient recovered from the ILD after 33 days of prednisolone administration. The dose of prednisolone was reduced to 20 mg, and the patient was discharged. The administration of prednisolone continued after the hospital discharge, and no sequela was subsequently recognized.

## Discussion

This EPPV study provides safety information for a 6-month period after the administration of encorafenib and binimetinib in Japanese patients with advanced *BRAF* V600E-mutated colorectal cancer, which is the first real-world safety data of the triplet and doublet regimens for colorectal cancer. In the phase III BEACON CRC study, gastrointestinal disorders, skin-related disorders, and pain-related disorders were the most commonly observed adverse events [5]. The profiles of ADRs in this EPPV were similar to those obtained in the BEACON CRC study.

EPPV is a pharmacovigilance activity of RMPs. Because ADRs collected in this EPPV depend on spontaneous reports from HCPs and patients, and because of the short follow-up period in some patients, such as those starting the therapy later in the EPPV period, the incidence of ADRs could be underestimated. In addition, this EPPV individually collected ADRs of encorafenib and those of binimetinib, but irrespectively of regimens, resulting in difficulty in distinguishing between ADRs of the triplet regimen and those of the doublet regimen. In this EPPV, ADRs were graded according to the IME list, but not to the National Cancer Institute Common Terminology Criteria for Adverse Events (CTCAE), which is generally used in most clinical trials. Therefore, ADRs in the IME list, such as serous retinal detachment, were always graded as SADRs regardless if their severity was mild (grade 1) or moderate (grade 2) according to CTCAE. These limitations may affect the discussion presented below.

Eye disorders are important risk factors that are associated with the use of encorafenib and binimetinib. Preclinical and clinical data have suggested that blocking the MAP kinase signaling cascade by *BRAF* inhibitors such as encorafenib, and MEK inhibitors such as binimetinib, can block not only growth suppression of tumor cells, but can also lead to a disturbance in the retinal pigment epithelium [10]. Eye disorders were observed in clinical trials with encorafenib and binimetinib for advanced colorectal cancer and for advanced melanoma [5, 8, 9]. In this EPPV, 22 eye disorders were observed as SADRs according to the IME list, but in the initial spontaneous reports, only one of them was graded as a SADR and the other 21 were non-SADRs. More than half of the eye disorders developed within 1 week after the administration started, which was similar to the findings in an expanded access program of the triplet regimen for advanced *BRAF* V600E-mutated colorectal cancer in Japan. The 22 cases of SADRs with eye disorders had mostly recovered or had not worsened with appropriate dose reduction, interruption, and discontinuation of the treatment. The product package insert recommends that treatment with encorafenib and binimetinib should be discontinued when any grade 4 eye disorders are recognized (Online Resource 3). With grade 1 retinal disorders or uveitis, continuation of treatment with encorafenib and binimetinib is allowed, while for grade 2–3 retinal disorders or uveitis, the treatment should be withheld until the grade decreases. Regarding uveitis, the differential diagnosis by ophthalmological examinations is critical to determine appropriate treatments that are different for non-infectious uveitis, infectious uveitis, and masquerade syndrome. When any grade retinal vein occlusion is recognized, encorafenib and binimetinib treatment should be discontinued. For any other grade 3 eye disorder, the treatment should be withheld until the disorder resolves to grade 1 or less. Because these eye disorders without appropriate management sometimes result in severe visual impairments, it is important to carefully monitor eye conditions from the initiation of treatment. Patients may not recognize the symptoms by themselves, and therefore, consultation with ophthalmologists should be considered promptly to appropriately manage the eye disorders.

ILD is often associated with the use of antineoplastic drugs, and is recognized as a noticeable ADR because of possible severe clinical outcomes. In this EPPV, one patient developed a serious ILD, which was resolved by standard treatments. The physician could not rule out a possible relationship of the ILD with encorafenib, binimetinib, or cetuximab. In Japan, ILD is listed as a serious ADR in the package insert of cetuximab and as an important potential risk in the RMPs of encorafenib and binimetinib, and therefore, the risk for ILD should be carefully monitored during treatment with the triplet and doublet regimens.

Currently, another prospective post-marketing surveillance study is ongoing in Japan as a pharmacovigilance activity of the triplet and double regimens for *BRAF* V600E-mutated colorectal cancer patients to assess the safety and efficacy of each regimen. The results of this study should also be considered to develop a treatment strategy and to clarify the safety profile for advanced *BRAF* V600E-mutated colorectal cancer.

## Conclusion

The incidence of ADRs associated with the triplet regimen of encorafenib, binimetinib, and cetuximab and the doublet regimen of encorafenib and cetuximab for 6 months after the approval was comparable to that observed in the phase III BEACON CRC study. SADRs that were not described in the package inserts were occasionally observed, but no new safety concerns were identified, although these results should be considered with caution, since this surveillance depended on spontaneous reports. Appropriate treatments following the latest package inserts and guidelines are vital, and the results of this EPPV and ongoing post-marketing surveillance studies may increase their effectiveness.

## Supplementary Information

Below is the link to the electronic supplementary material.

Supplementary file1 (DOCX 109 KB)

## Data Availability

Qualified researchers may request Ono Pharmaceutical Co., Ltd. to disclose individual patient-level data from clinical studies through the following website: https://www.clinicalstudydatarequest.com/. For more information on Ono Pharmaceutical Co., Ltd.'s Policy for the Disclosure of Clinical Study Data, please see the following website: https://www.ono-pharma.com/en/company/policies/clinical_trial_data_transparency_policy.html.
