# Supplementary material for: Encorafenib, binimetinib, and cetuximab in BRAF V600E–mutated colorectal cancer: an early post-marketing phase vigilance study
Source: Int J Clin Oncol. 2022 Nov 10;28(1):139–44. doi: 10.1007/s10147-022-02264-z (PMC9823055; doi:10.1007/s10147-022-02264-z)

**Encorafenib, Binimetinib, and Cetuximab in *BRAF* V600E–Mutated Colorectal Cancer: An Early Post-Marketing Phase Vigilance Study**

International Journal of Clinical Oncology

Hidenori Sakata, Maki Murase, Takeshi Kato, Kensei Yamaguchi, Kenichi Sugihara, Shigenobu Suzuki, and Takayuki Yoshino

Corresponding author:

Hidenori Sakata, M.Pharm

Department of Pharmacovigilance, Ono Pharmaceutical Co. Ltd.

Email: h.sakata@ono.co.jp

**Online Resource 1** Abbreviations

ADR Adverse drug reaction

BEACON CRC An open-label, international, phase III study

CTCAE Common Terminology Criteria for Adverse Events

EGFR Epidermal growth factor receptor

EPPV Early post-marketing phase vigilance

HCP Healthcare professional

ILD Interstitial lung disease

IME Important medical event

MEK Mitogen-activated protein kinase

RMP Risk management plan

SADR Serious adverse drug reaction

**Online Resource 2** Important risks of encorafenib and binimetinib in Japan

|  | **Encorafenib** | **Binimetinib** |
| --- | --- | --- |
| **Important identified risks** | - Cutaneous malignancies - Eye disorders - Palmar-plantar erythrodysesthesia syndrome | - Cardiac dysfunction - Hypertension - Rhabdomyolysis - Hepatic dysfunction - Eye disorders - Hemorrhage |
| **Important potential risks** | - Secondary malignancies other than cutaneous malignancies - Cardiac dysfunction - Hypertension - Rhabdomyolysis - Hepatic dysfunction - Hemorrhage - Interstitial lung disease - Renal impairment - QT prolongation - Drug-drug interactions in concomitant use with moderate or potent CYP3A inhibitors - Embryo-fetal toxicity | - Interstitial lung disease - Venous thromboembolism - Renal impairment - QT prolongation - Safety in patients with hepatic impairment - Embryo-fetal toxicity |
| **Important missing information** | Not applicable | Not applicable |

**Online Resource 3** Recommended dose modifications at the onset of eye disorders


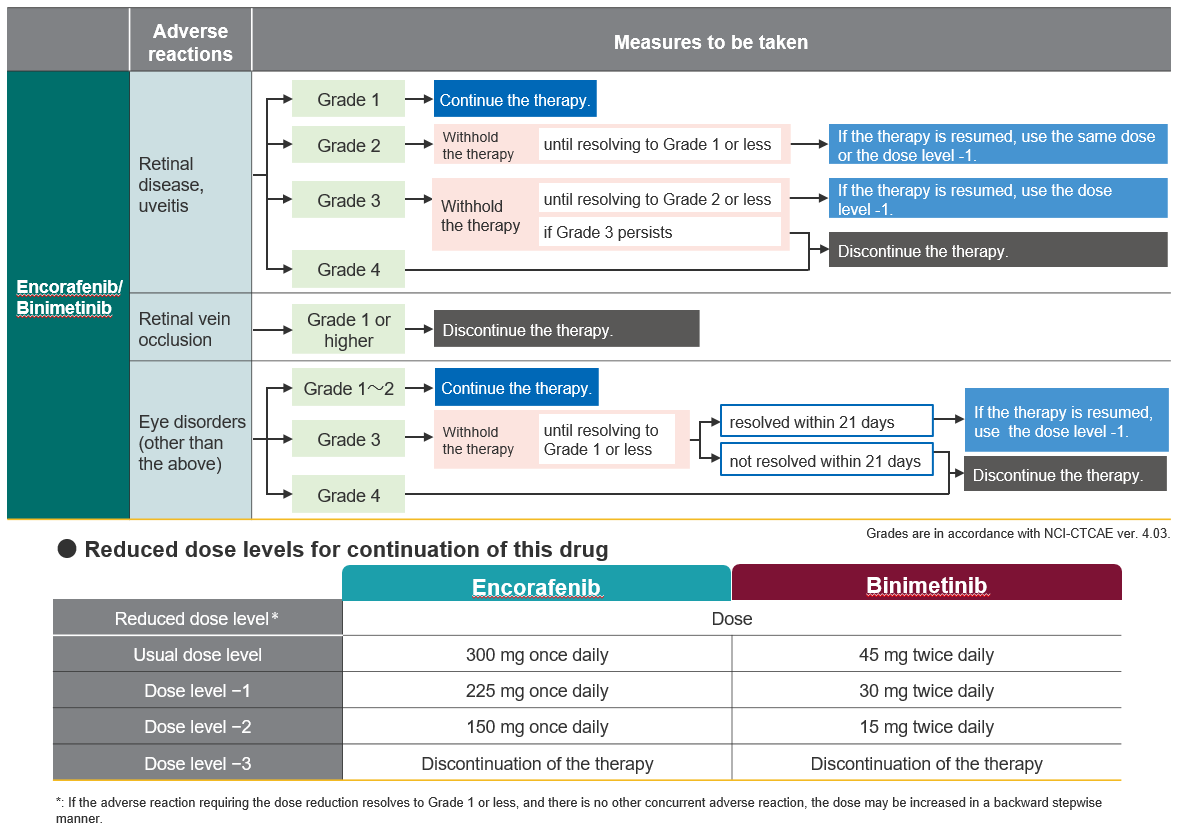

Supplement: Supplementary file 1 — Supplementary file1 (DOCX 109 KB) [file 10147_2022_2264_MOESM1_ESM.docx]
